# Supplementary material for: An Outbreak of Limping Syndrome Associated with Feline Calicivirus
Source: Animals (Basel). 2023 May 26;13(11):1778. doi: 10.3390/ani13111778 (PMC10251824; doi:10.3390/ani13111778)

Figure S1: Unrooted phylogenetic tree based on the complete nucleotide sequence of FCV strains detected in this study and cognate strains retrieved from GenBank database. The Maximum Likelihood method and general time-reversible model (six parameters) with a gamma distribution and invariable sites were used for the phylogeny. A total of 1000 bootstrap replicates were used to estimate the robustness of the individual nodes on the phylogenetic tree. Bootstrap values greater than 75% were indicated. Black arrows indicate strains detected in this study. White arrows with black outline indicate limping strain retrieved from GenBank databases. Numbers of nucleotide substitutions are indicated by the scale bar. Abbreviations: LIM=limping pathotype; VAC= vaccine; ORD= oral respiratory disease; ENT=enteric pathotype; VSD= virulent systemic disease; CYS=cystitis pathotype; ABO=abortion pathotype; ASY=asymptomatic pathotype; UNK=unknown pathotype.

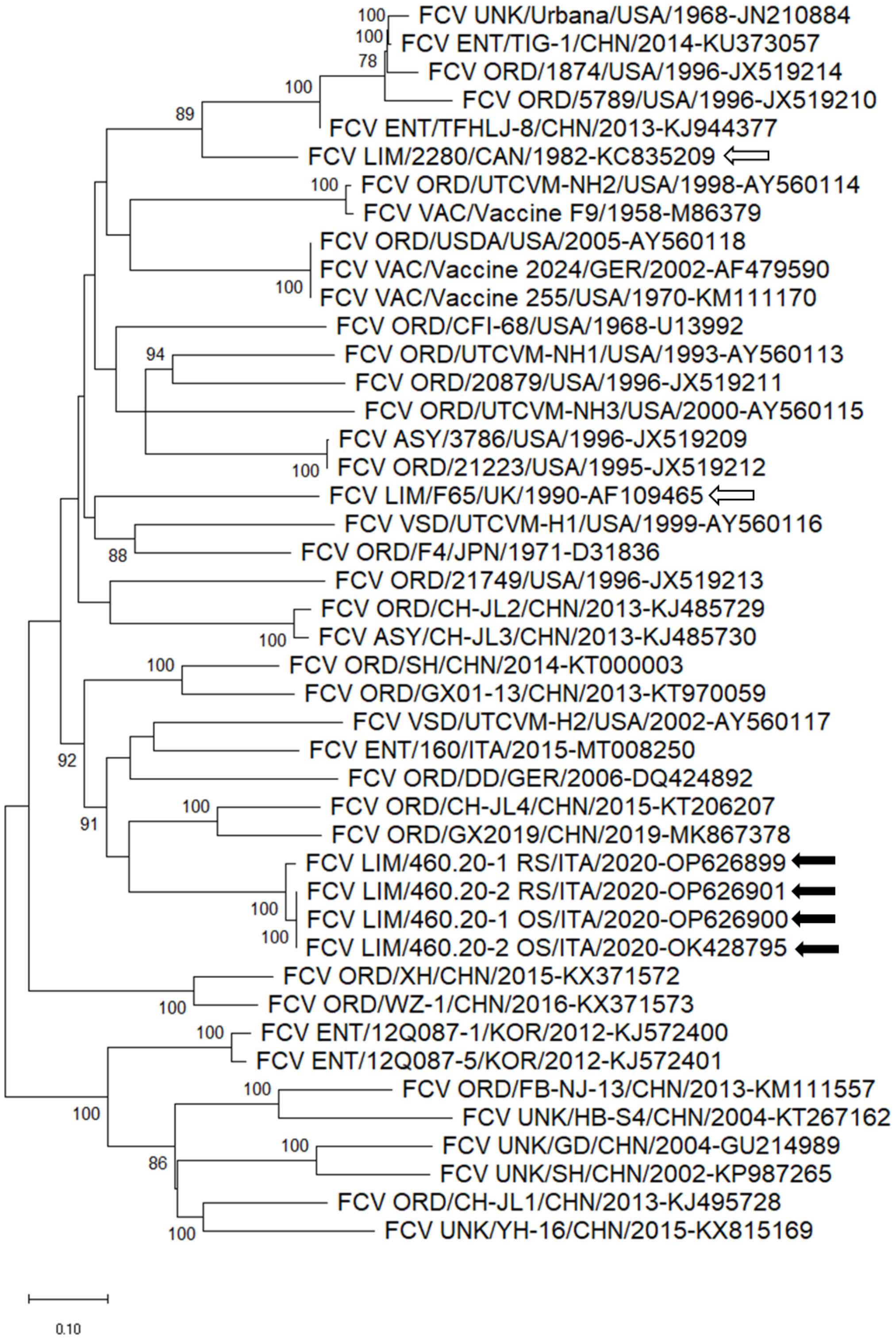

Supplement: Supplementary file 1 [file animals-13-01778-s001.zip › animals-2384092-supplementary.pdf]
